# Domain Architecture of the Nonreceptor Tyrosine Kinase Ack1

**DOI:** 10.3390/cells12060900

**Published:** 2023-03-15

**Authors:** Yagmur Kan, YiTing Paung, Markus A. Seeliger, W. Todd Miller

**Affiliations:** 1 Department of Physiology and Biophysics, School of Medicine, Stony Brook University, Stony Brook, NY 11794-8661, USA; 2 Department of Pharmacology, School of Medicine, Stony Brook University, Stony Brook, NY 11794-8661, USA; 3 Department of Veterans Affairs Medical Center, Northport, NY 11768-2200, USA

**Keywords:** nonreceptor tyrosine kinase, Ack1, SAM domain, activated Cdc42-associated kinase, ubiquitin-associated domain

## Abstract

The nonreceptor tyrosine kinase (NRTK) Ack1 comprises a distinct arrangement of non-catalytic modules. Its SH3 domain has a C-terminal to the kinase domain (SH1), in contrast to the typical SH3-SH2-SH1 layout in NRTKs. The Ack1 is the only protein that shares a region of high homology to the tumor suppressor protein Mig6, a modulator of EGFR. The vertebrate Acks make up the only tyrosine kinase (TK) family known to carry a UBA domain. The GTPase binding and SAM domains are also uncommon in the NRTKs. In addition to being a downstream effector of receptor tyrosine kinases (RTKs) and integrins, Ack1 can act as an epigenetic regulator, modulate the degradation of the epidermal growth factor receptor (EGFR), confer drug resistance, and mediate the progression of hormone-sensitive tumors. In this review, we discuss the domain architecture of Ack1 in relation to other protein kinases that possess such defined regulatory domains.

## 1. Introduction

Activated Cdc42-associated kinase (Ack1, TNK2) is a nonreceptor tyrosine kinase (NRTK) that belongs to the Ack family of kinases. The gene encoding Ack1, TNK2, is located on chromosome 3q29 in humans and is activated in response to growth factors and integrin-mediated signaling pathways [1]. Activated Ack1 is recruited to receptor tyrosine kinases (RTKs) such as platelet-derived growth factor receptor (PDGFR) [2], insulin receptor (IR), Mer [3], and epidermal growth factor receptor (EGFR) [4]. The Ack1 stands out among the other NRTKs because of its unusual domain composition. The Ack1 comprises an N-terminal sterile alpha motif (SAM) domain [2], a catalytic domain, followed by a Src homology 3 (SH3) domain, and a Cdc42/Rac-interactive domain (CRIB) [5]. The Ack1 is the only tyrosine kinase (TK) with a CRIB domain that specifically binds to the small GTPase Cdc42. The C-terminal region of Ack1 has other domains atypical for tyrosine kinases, such as a clathrin-binding (CB) motif [6], a proline-rich carboxyl-tail, a region that shares high homology with Mig6 protein (MHR) [2], and a ubiquitin association (UBA) domain.

Given the unique domain composition of Ack1 among NRTKs, this review aims to analyze the distinct features and domain architecture of Ack1 considering the current knowledge of other proteins that possess these domains. Recent reviews have extensively discussed the regulation and signaling pathways of Ack1 [7], its involvement in cancer [8], and the latest efforts to inhibit Ack1 as a therapeutic target [9]. Therefore, we will examine these topics only briefly.

### 1.1. Ack Family Kinases

**Figure 1:**
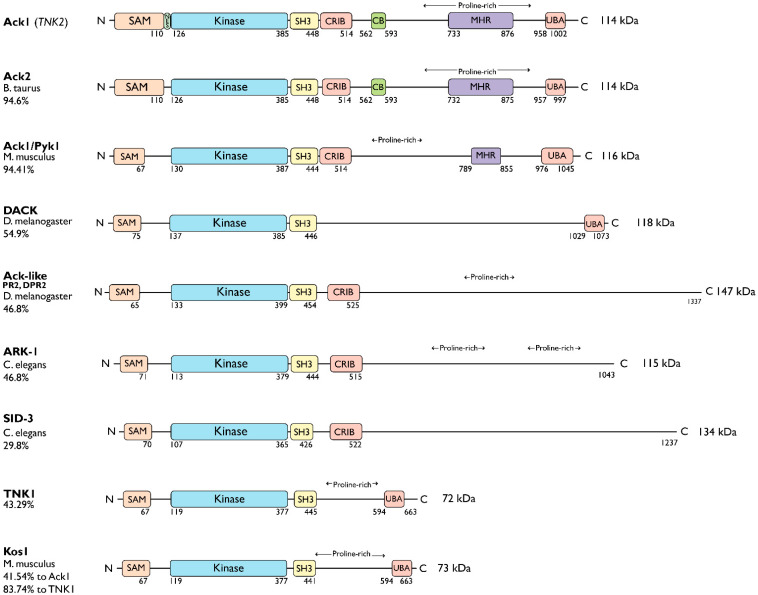
Ack family kinases. Linear representation of Ack family members. The residue numbers below each domain depict the boundaries of the domains retrieved from the UniProtKB [10]. SAM, sterile alpha motif; NES, nuclear export signal; CRIB, Cdc42, and Rac-interactive binding (CRIB) domain; CB, clathrin-binding motif; proline-rich region; MHR, Mig6 homology region; UBA, ubiquitin-associated domain. The sequence identity between each member and human Ack1 (or TNK1) is indicated to the left of the linear diagram as percentage identity. UniProtKB IDs used to depict the domain arrangements and to determine percent sequence identity are human Ack1, Q07912; bovine Ack2, Q17R13; mouse Ack1/Pyk1, O54967; fruit fly DACK, Q9VZI2; fruit fly Ack-like/DPR2/PR2, Q9I7F7; worm Ark-1, G5EBZ8; worm sid-3, Q10925; human TNK1, Q13470; and mouse TNK1/Kos1, Q99ML2.

The Ack kinase family consists of Ack1 and TNK1 in humans, their homologs Ark-1 and sid-3 in *Caenorhabditis elegans*, Ack-like and DACK in *Drosophila*, bovine Ack2 and Ack1/Pyk1 and Kos1 in mice (Figure 1). All members of the Ack kinase family possess a SAM domain, which is uncharacteristic of nonreceptor tyrosine kinases, and an SH3 domain C-terminal to the catalytic domain. Notably, Acks are the only NRTKs known to have an SH3 domain at the C-terminus of their kinase domain.

**Figure 2:**
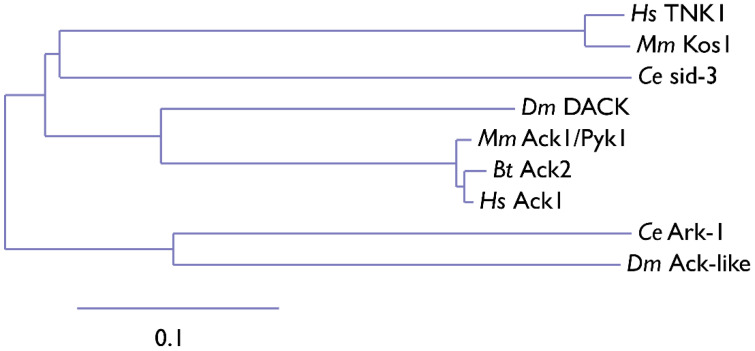
Phylogenetic tree of the Ack1 family. Ce, *Caenorhabditis elegans* (nematode); Dm, *Drosophila melanogaster* (fruit fly); Bt, *Bos taurus* (cow); Hs, *Homo sapiens* (human); Mm, *Mus musculus* (mouse). The scale indicates the rate of amino acid substitution per residue. The amino acid sequences of the kinase domains of Ack family proteins from *C. elegans* (sid-3, Ark-1), *D. melanogaster* (DACK, Ack-like), *B. taurus* (Ack2), *M. musculus* (Ack1, Kos1), and *H. sapiens* (Ack1, TNK1) were used for phylogenetic analysis. The phylogenetic tree was manually compiled using MAFFT, Gblocks, PhyML, and TreeDyn at Phylogeny.fr [17].

The TNK1 is encoded by the human chromosome 17p13.1 [11]. Similar to Ack1, TNK1 also contains a UBA domain with a high affinity for polyubiquitin [12]. However, it lacks the CB, CRIB, and MHR domains. The murine homolog of TNK1, Kos1 [13], is located in the middle region of chromosome 11 in mice, which corresponds to chromosome 17p13.1 in humans [12,14], and it is thought to be a splice variant of TNK1 [13]. The Kos1 knockout mice develop spontaneous tumors, suggestive of a role in cancer development [15]. Phylogenetic analysis of Ack kinase domains shows that the closest relative of TNK1 and Kos1 is *C. elegans* sid-3. The Sid-3 kinase is more closely related to human Ack1 than to worm ARK-1, an Ack-related kinase (Figure 2). Similar to its human homolog, Ark-1 downregulates let-23, the *C. elegans* homolog of EGFR [16]. Signaling events involving the downregulation of let-23 have been proposed to be mediated by sem-5, the *C. elegans* homolog of Grb2.

The murine homolog of Ack1 was identified as a Grb2-binding protein and was initially named proline-rich tyrosine kinase Pyk1 [18]. Similar to its human counterpart, mouse Ack1 is highly expressed in the developing and adult brain [18]. The expression of Ack1 is upregulated with increased neural activity, suggesting a role for Ack1 in mouse synaptic function, plasticity, and brain development.

Similarly, as a splice variant of human Ack1, the bovine homolog of Ack1, Ack2 [19], retains the CRIB domain and CB motif following its SH3 domain. The Ack2 shares ~95% sequence identity with Ack1. Overexpression of Ack2 in NIH3T3 cells severely impairs cell growth [20]. In fruit flies (*D. melanogaster*), DACK and Ack-like (DPR2, PR2) are two Ack family kinases that are also effectors of the small GTPase Cdc42 [21,22,23]. Compared to Ack-like, DACK is more closely related to Ack1.

The worm ARK-1 and SID-3, and fly DACK and Ack-like, only have the CRIB domain following the SH3 domain and lack the other domains present in Ack1. The ARK-1 acts as a negative regulator of EGFR signaling in *C. elegans* [24] paralleling its human counterpart. While ARK-1 suppresses cell division in embryos [16,25], SID-3 modulates the efficient import of dsRNA in worms [26]. Human Ack1 is known to associate with endocytic vesicles, which are also involved in dsRNA transport.

Overall, the Ack family kinases regulate many important biological activities, including cell adhesion, apoptosis, tumor cell survival, proliferation, T-cell activation, DNA repair, synapse formation, and membrane trafficking.

### 1.2. Mechanism of Action

**Figure 3:**
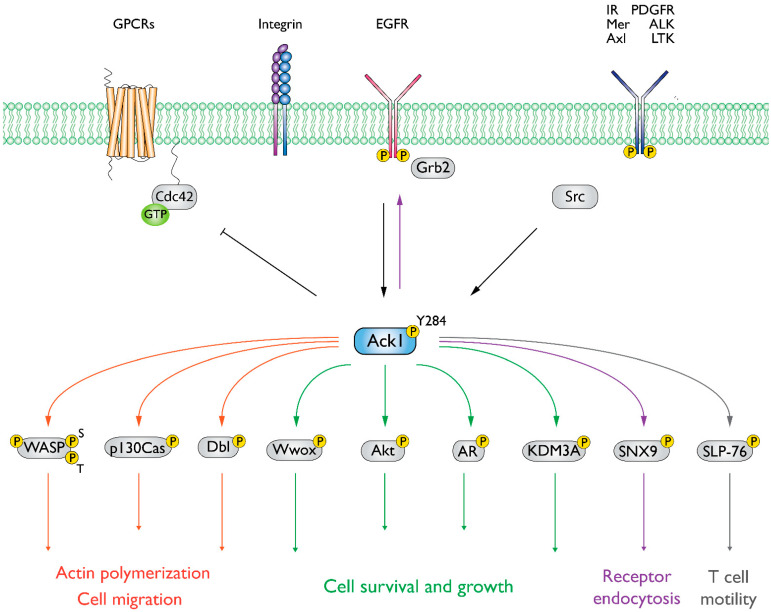
Summary of Ack1-mediated signaling events. Ack1 is activated in response to growth factors, G-protein coupled receptors (GPCRs), and integrin-mediated cell adhesion and is recruited to activated RTKs such as platelet-derived growth factor receptor (PDGFR), insulin receptor (IR), Mer, and epidermal growth factor receptor (EGFR) [2,3,25,28,31]. In cancer cells, Ack1 phosphorylates the tumor suppressor Wwox [3] and facilitates uncontrolled activation of pro-proliferative signals (Akt and AR). A detailed discussion of the signaling pathways can be found in the study by Fox et al. [7].

The Ack1 is activated downstream of a broad range of receptors such as integrins, muscarinic M3 receptors, PDGFR, Axl, IR, Mer, Trk, and EGFR, as summarized in Figure 3 [2,27,28,29]. Similar to the Src family kinases, the activation of Ack1 involves autophosphorylation (at Y284). In vitro studies using purified Ack1 have demonstrated that autophosphorylation moderately enhances its activity and that phosphorylation is regulated by dimerization via the SAM domain. Phosphorylation of the activation loop does not have a significant effect on the substrate or ATP binding [30].

The Ack1 adopts a Src-like active conformation in its phosphorylated (Protein Data Bank [32], PDB: 1U4D) and unphosphorylated states (PDB: 1U46). The activation loop of both structures is well-ordered, with R247 forming hydrogen bonds with the backbone carbonyl of P278 and N281, suggesting that both structures are in active conformation. Typically, the activation loop in NRTKs adopts an autoinhibitory conformation in the unphosphorylated state. However, in Ack1, M274 of the activation loop extends outside the loop to position the hydrogen interactions between the loop and C-lobe, facilitating the stabilization of the unphosphorylated activation loop. Most TKs have a small residue at this position; a rather large hydrophobic M274, which is conserved throughout the Ack family, stabilizes the unphosphorylated state of the activation loop in Ack1.

The catalytic activity of Ack1 increases 20- to 30-fold by a head-to-head symmetric dimerization. This dimerization-mediated activation model is further supported by evidence that phosphorylation has a minimal effect on activity [33]. Notably, Ack1 does not undergo significant structural changes upon binding to nucleotides. The structure of Ack1 bound to the nucleotide analog, AMP-PCP (PDB: 1U54), is comparable to nucleotide-free Ack1 in both phosphorylated (PDB: 1U4D) and unphosphorylated form (PDB: 1U46) with an RMSD of 0.5 Å overall Cα pairs [30].

### 1.3. Emerging Roles in Signaling Pathways

Other than its established proto-oncogenic roles, recent work has provided evidence for the involvement of Ack1 in (1) neural signaling and (2) immune signaling pathways.

#### 1.3.1. Role of Ack1 in Neural Signaling Pathways

The Ack1 is expressed ubiquitously in all tissues in humans, with especially high expression in neurons of the developing and adult brain, at both the mRNA and protein levels. In neurons, Ack1 plays a role in neurotrophin signaling cascades [29], which are mainly mediated by nerve growth factors, neurotrophins, and Trk receptors that regulate neuronal development. The Ack1 interacts with Trk receptors and is tyrosine phosphorylated in response to neurotrophins. The Ack1 overexpression in primary neuronal cells induces neurite outgrowth and promotes branching in neurotrophin-treated neuronal cells. In addition, Ack1 is involved in the Ras-GRF1 signaling cascade in neuronal cells and mediates calcium influx in the brain [34].

Another involvement of Ack1 in neural signaling pathways is through dopamine transporters. Dopamine is a vital regulator of physiological and behavioral pathways such as voluntary motor movement and reward [35]. The dopamine transporter protein DAT controls dopamine neurotransmission and facilitates its clearance from synapses, resulting in the termination of dopamine signaling. The DAT is regulated and internalized via clathrin-mediated endocytosis. A key modulator of DAT endocytosis is the activation of protein kinase C (PKC), which promotes the internalization of DAT. The Ack1 works antagonistically to PKC and prevents the endocytosis of DAT, sustaining DAT in the plasma membrane. This effect is overcome by PKC activation, which inactivates Ack1 [36]. A reduction in DAT density in the plasma membrane is implicated in Parkinson’s disease, which emphasizes the importance of endocytic regulation of DAT. Further research is needed to address Ack1’s connection to dopamine signaling and to identify the upstream and downstream modulators involved.

#### 1.3.2. Role of Ack1 in Immune Signaling Pathways

The second emerging area of research involving Ack1 is in immune cell signaling cascades. The Ack1 expression is associated with immune cell infiltration and immunomodulators, which are pronounced in cancer immunity. In lung cancer, Ack1 mRNA levels are inversely correlated with the infiltration levels of B cells, CD8+ T-cells, CD4+ T-cells, macrophages, neutrophils, and dendritic cells [37]. In addition to immune cell infiltration, dysregulation of Ack1 is linked to abnormal apoptotic activity [38], which is crucial for the elimination of cytotoxins and self-antigens.

Silencing of the TNK2 gene, which encodes Ack1, leads to the activation of several immune-related signaling pathways, including T-cell receptor (TCR), chemokine, JAK-STAT, and Toll-like receptor (TLR) signaling pathways. Specifically, Ack1 regulates the activation of TLR signaling pathways such as TLR4, TLR7, and TLR9, which mediate inflammation and autoimmunity by controlling macrophages and dendritic cells [39]. In macrophages, Ack1 overexpression promotes the activation of the TLR4, TLR7, and TLR9 pathways, while Ack1 knockdown inhibits their activation. Pharmacological inhibition of Ack1 activity reduces TLR-mediated activation of macrophages, relieving endotoxic shock and lupus symptoms in mouse models [39]. These mouse models are the first to document the role of Ack1 in inflammation and autoimmunity, suggesting a new avenue where Ack1 inhibition could serve as a therapeutic.

Furthermore, Ack1 mediates early T-cell activation events in a process involving the SAM domain of Ack1. The Ack1 interacts with SLP-76, a protein known to play a crucial role in signal transmission to the transcriptional machinery [40]. Both SLP-76 and Ack1 interact through a SAM–SAM interaction where the SAM domain of Ack1 binds to the SAM domain of SLP-76. The Ack1 phosphorylates SLP-76 at Y113, Y128, and Y145, and phosphorylation of these residues is required for the TCR activation [41,42].

### 1.4. Ack1 Substrates

**Figure 4:**
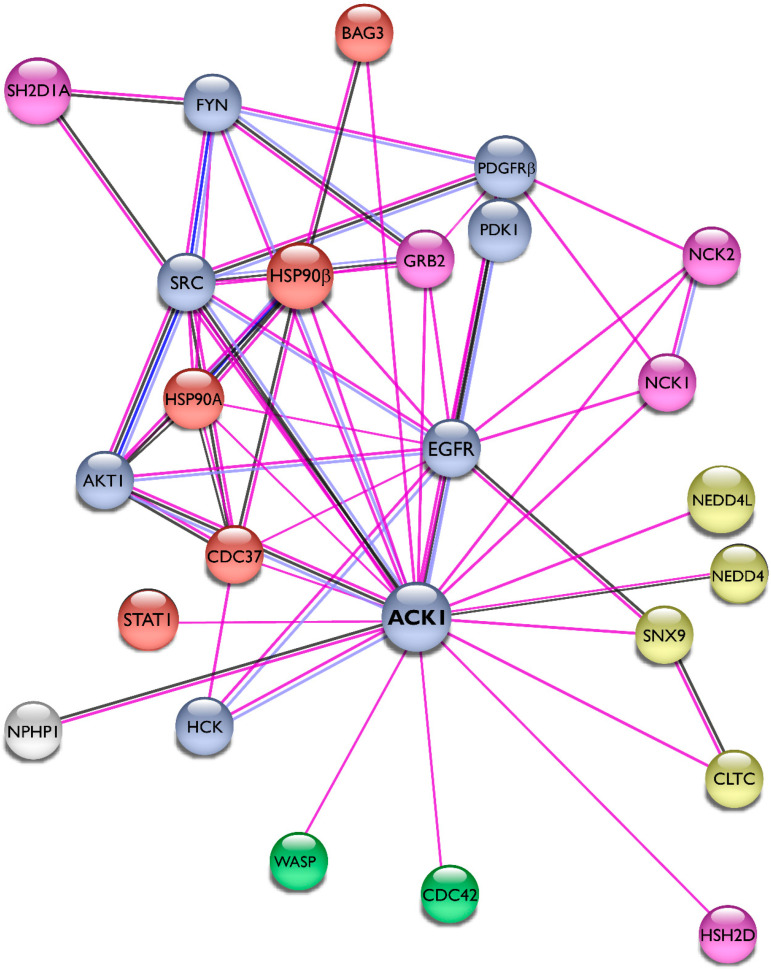
Interaction network of Ack1. Using the STRING database [43], we mapped the Ack1-interacting proteins from Mahajan [44]. Lines are color-coded according to the nature of the association, which is based on experimental evidence (pink), co-expression (dark blue), or protein homology (gray). Nodes are color-coded by function (protein kinases, light blue; adaptor proteins, pink; heat shock protein complex, orange) or signaling networks (actin polymerization, green; receptor endocytosis, yellow).

We summarized the protein–protein interactions of Ack1 as a STRING network [43] and included only experimentally validated interactions representing both physical and functional associations in Figure 4. The interaction network of Ack1 includes protein kinases (EGFR, PDGFR, Akt, Src, Fyn, Hck, PDK1), adaptor molecules (Grb2, HSH2, NCKs), Heat shock protein 90 and its co-chaperones (HSP90, Cdc37, Bag3), proteins regulating actin dynamics (Cdc42, WASP), and proteins involved in vesicle dynamics (CLTC, SNX9, NEDD4). More details on interacting proteins and substrates of Ack1 are described in the review by Mahajan and Mahajan [44].

### 1.5. Ack1 Involvement in Disease

The role of Ack1 in different diseases has been extensively studied and reviewed by Mahajan [8] and Owen [7]. Generally, Ack1 involvement in cancer and other diseases occurs at one of three different levels: genomic (amplification and mutations), transcriptional (overexpression), and post-transcriptional (downregulation of tumor suppressors).

#### 1.5.1. Genomic Level

The gene encoding Ack1, TNK2, is amplified in human cancers, including prostate, breast, esophageal, lung, ovarian, and pancreatic cancers, with the highest gene amplification being 9% in ovarian and 14% in lung primary tumors [45]. The Ack1 activity is elevated in multiple cancer cell lines, including breast [46], colon [47,48], prostate [3], gastric [49,50], ovarian [51], and liver cancers [52]. In gastric cancer, the DNA copy numbers of TNK2 were significantly higher compared to those of normal gastric tissues [49,50]. Existing data shows that Ack1 is amplified or mutated in breast, ovarian [53], colorectal cancers [54], esophageal squamous cell carcinoma [55], non-small-cell lung cancer [56], osteosarcoma [57], and chronic myelomonocytic leukemia [58].

#### 1.5.2. Transcriptional Level

Overexpression of TNK2 has been identified in 42% of aggressive lung tumors, with elevated RNA levels ranging from 6- to 35-fold [38]. In metastatic hormone-refractory prostate tumors, the overexpression is more prevalent, with 77% of the tissues exhibiting elevated RNA levels (5- to > 100-fold). In triple-negative breast cancer cell lines, Ack1 expression correlates with high proliferation, invasion, and colony-forming abilities [59]. Overexpression of Ack1 also promotes hepatocellular carcinoma progression [52], which is associated with tumor recurrence and poor survival [60].

In addition to cancer, the transcriptional dysregulation of Ack1 has been implicated in several neural disorders. For example, overexpression of Ack1/EGFR has been linked to epilepsy [61]. A homozygous missense variant of Ack1, V638M, has been documented in a family with infantile-onset autosomal recessive epilepsy and intellectual disability. The variant resulted in overexpression of Ack1 owing to its improper degradation. Further research is needed to fully understand gain-of-function mutations in Ack1 and their implications for perturbed signaling in neural diseases.

#### 1.5.3. Post-Transcriptional Level

Another mechanism by which Ack1 is involved in cancer progression is by negatively regulating tumor suppressors such as Wwox and positively regulating pro-survival signaling pathways (reviewed in [31]). Through Akt phosphorylation and activation of the PI3K pathway, Ack1 regulates the expression of 147 different proteins associated with metastasis and epithelial-mesenchymal transition (EMT) in gastric cancer [50]. The activation of Akt is also implicated in glioblastoma multiforme through the elevation of Ack1 phosphorylation and increased PDGFR signaling [62].

A novel role of Ack1 as an epigenetic regulator has been reported in castration-resistant prostate cancer (CRPC). The Ack1 phosphorylates histones (H4) upstream of the androgen receptor (AR) transcription start site, promoting its expression in the absence of androgen. Consequently, through epigenetic regulation and tyrosine phosphorylation, Ack1 is a key player in dysregulated signaling events in androgen-independent prostate cancer [63,64,65,66,67]. 

### 1.6. Ack1-Mediated Drug Resistance

Several lines of evidence have demonstrated Ack1-mediated drug resistance in hormone-dependent cancers. In breast cancer, Ack1 drives the expression of the HOXA oncogene, making cells resistant to tamoxifen therapy [4]. In HER2 overexpressing tumors, Ack1 is activated downstream of HER2 and acts as an indirect epigenetic regulator. The Ack1 phosphorylates the histone demethylase KDM3A, which in turn promotes estrogen receptor (ER)-driven transcription of HOXA in the absence of estrogen. In this manner, Ack1-driven transcription of HOXA, a critical mediator of breast cancer progression, overrides treatment with the ER antagonist tamoxifen.

The Ack1 serves as a prognostic marker in numerous types of cancer, and its overexpression or hyperphosphorylation tends to correlate with poor prognosis. As reviewed by Mahajan and Mahajan [31], Ack1 Y284 is a biomarker for prostate cancer disease progression and negatively correlates with survival [3,63,65,68,69,70].

In contrast to its hyperactivation or overexpression, Ack1 is downregulated in vemurafenib-resistant melanoma with activated EGFR [71]. Downregulation of Ack1 in these cells causes a decrease in EGFR degradation, as evidenced by in vitro and in vivo melanoma models. This presents another post-transcriptional mechanism through which Ack1 contributes to drug resistance.

### 1.7. Domain Localization of Cancer-Associated Mutations

**Figure 5:**
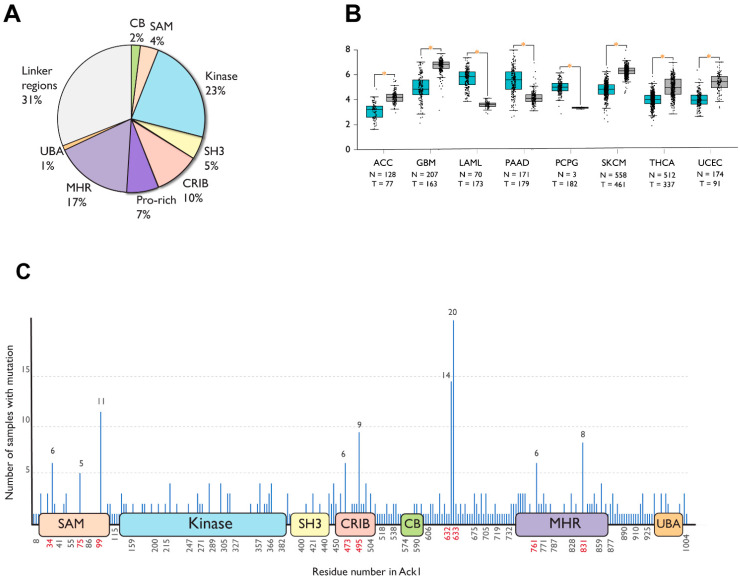
Cancer-associated mutations in Ack1 in the COSMIC database. (**A**) Distribution of nonsynonymous mutations. SAM, sterile alpha motif; CRIB, Cdc42, and Rac-interactive binding (CRIB) domain; CB, clathrin-binding motif; proline-rich region; MHR, Mig6 homology region; UBA, ubiquitin-associated domain (**B**) Ack1 expression in normal (blue) vs. tumor (gray) tissues. ACC: adrenocortical carcinoma, GBM: glioblastoma multiforme, LAML: acute myeloid leukemia, PAAD: pancreatic adenocarcinoma, PCPG: pheochromocytoma, SKCM: skin cutaneous melanoma, THCA: thyroid carcinoma, UCEC: uterine corpus endometrial carcinoma. (**C**) Locations of cancer-associated mutations outlined in the linear structure of Ack1. SAM, sterile alpha motif; NES, nuclear export signal; CRIB, Cdc42, and Rac-interactive binding (CRIB) domain; clathrin-binding motif; proline-rich region; MHR, Mig6 homology region; UBA, ubiquitin-associated domain. Each horizontal line represents a mutation, with the number of mutations positively correlated with the length depicted on the *y*-axis. The *x*-axis shows the location of the mutations. Highly mutated residues are shown in red. Note that the numbering convention used in COSMIC is based on an isoform different from the canonical one (UniProtKB: A0A5F9ZHL4), and that the figure uses the isoform 1 (UniProtKB: Q07912) convention.

To date, 863 different somatic mutations have been documented in the Catalogue of Somatic Mutations in Cancer (COSMIC) [72,73]. Figure 5A summarizes the distribution of these mutations across the domains of Ack1. Most mutations occur in the linker regions (31%), the kinase domain (23%), followed by the MHR (17%). 

Similar to COSMIC, the Gene Expression database of Normal and Tumor tissues 2 (GENT2) compiles gene expression patterns across different normal and tumor tissues from public databases [74]. As opposed to statistically significant upregulation in adrenocortical carcinoma, glioblastoma, skin cutaneous melanoma, thyroid, and endometrial cancer, TNK2 expression is downregulated in acute myeloid leukemia (AML), pancreatic cancer, and pheochromocytoma compared to the corresponding healthy tissues (Figure 5B). 

A significant portion (67 of 508) cancer-associated substitution or deletion mutations in Ack1 are localized to six different residues: R34, R99, D495, P633, P632, and Q831. The mutations involving P632 and P633 are truncating frameshift mutations residues that remove the C-terminal domains MHR and UBA, while R34 and R99 are located at the SAM domain (Figure 5C).

### 1.8. Ack1 Drug Development Efforts

The association of Ack1 with human disease has driven significant efforts toward the development of Ack1 inhibitors. The Ack1 was found to modulate sensitivity to tyrosine kinase inhibitors downstream of mutant CSF3R in patient-derived cell models of chronic neutrophilic leukemia and atypical chronic myeloid leukemia, making it a possible candidate for therapeutic intervention [75]. Indeed, Ack1 is an important therapeutic target for acute myeloid leukemia (AML) with constitutively active NRAS mutations [76,77] and in chronic myelomonocytic leukemia since these mutations are sensitive to inhibitors of Ack1 [58,78]. In juvenile myelomonocytic leukemia (JMML) and AML cells, Ack1 activates PTPN11, and PTPN11-mutant JMML and AML cells are all sensitive to Ack1 inhibition [78].

An enantiomer of piperazine-substituted chloropyrimidine, (R)-9b, has been identified as a potent inhibitor of Ack1 in vitro (IC_50_ = 56 nM, ^33^P HotSpot assay) and in vivo (IC_50_ < 2 μM, human cancer cell lines) and is stable in human plasma (half-life > 6h) [79]. In addition to (R)-9b, other potential inhibitors have been identified from existing clinical drugs [9,79,80]. Dasatinib is also a potent inhibitor of Ack1, which inhibits Ack1 with an IC_50_ of 1nM [81]. 

## 2. Ack Domain Structure

The Ack1 encompasses an N-terminal SAM domain, KD, CRIB domain, clathrin-binding motif, proline-rich motif, MHR motif, and UBA domain (Figure 1). In this review, we discuss the function and structure of Ack1 by examining its domain organization.

### 2.1. SAM Domain

Sterile alpha motifs (SAMs) are modules for protein–protein interactions and associate with SAM-containing proteins (homotypic and heterotypic) and non-SAM-containing structures such as proteins, DNA, and RNA. The SAM domains can form dimers and oligomers. In some proteins, the SAM domains inhibit receptor oligomerization and autoactivation, while dysregulation of the SAMs promotes ligand-free oligomerization, promoting oncogenicity. For example, the SAM domain of EphA2 RTK has an auto-inhibitory role and inhibits kinase activity by reducing receptor oligomerization [82]. In yeast, a subfamily of the SAM domains has a unique function: it perturbs clathrin binding by blocking the clathrin-binding motif and negatively regulates clathrin-mediated endocytosis [83]. In addition to their role in self-association, the SAM domains can also interact with membrane lipids and can be modified by phosphorylation, serving as kinase docking sites.

Mutations in the SAM domains have been linked to several diseases. For example, a gene fusion between the SAM domain of a transcription factor, and the receptor tyrosine kinase NTRK3 results in a chimeric oncogenic TK capable of transforming cells. Mutations in the SAM domain of this transcription factor-TK chimera block polymerization and kinase activation, abolishing the observed transforming phenotype in NIH 3T3 cells. 

#### 2.1.1. SAM Domain of Ack1: Structure and Function

The SAM domain of Ack1 serves multiple functions. First, it controls the localization of Ack1; deletion of the N-terminal SAM (∆SAM) domain abolishes Ack1 localization to the plasma membrane [84]. Second, the SAM domain regulates activity; ∆SAM causes a 2-fold decrease in autophosphorylation of full-length Ack1 WT, suggesting that the SAM domain is important for maintaining full kinase activity. This effect of ∆SAM correlates with its role in dimer formation as ∆SAM weakens the dimerization of Ack1 [84]. Additionally, the SAM domain of Ack1 is suggested to play a role in its lysosomal degradation as the deletion of SAM dramatically reduces Ack1 ubiquitination [85].

**Figure 6:**
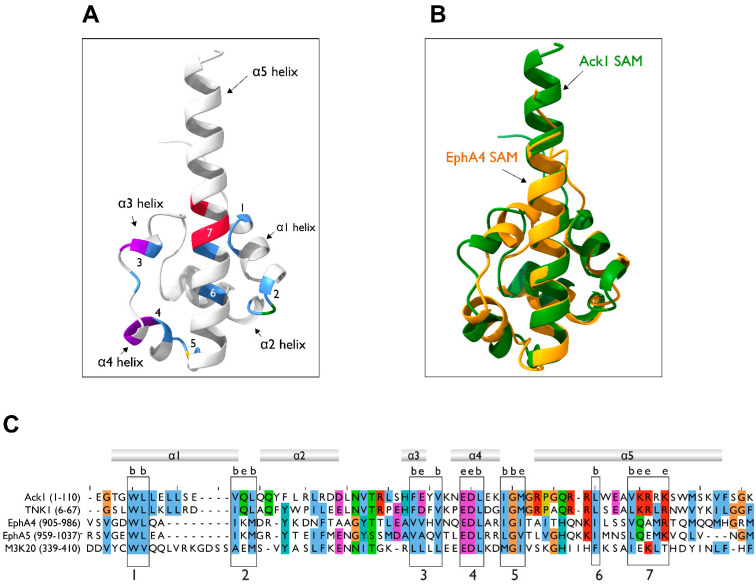
Homology modeling of the SAM domain. (**A**) AlphaFold model of the Ack1 SAM domain. The Ack1 SAM domain is predicted to adopt a typical SAM domain fold, with four short α helices, α1-α4, and a long C-terminal α-helix, α5. Numbers denote the location of the boxes in panel (**C**). (**B**) Superimposition of the predicted Ack1 SAM domain and the EphA4 SAM domain (PDB: 1B0X). (**C**) Multiple sequence alignment of the SAM domains of human Ack1 and TNK1, EphA4, EphA5, and ZAK (M3K20) kinases. The numbers in parentheses denote the residues used as the inputs. Consensus sequences for SAM are highlighted in numbered boxes. The letters b and e denote the residues predicted to be buried or exposed, respectively.

The three-dimensional structure of the Ack1 SAM domain has not been determined. However, based on sequence similarity to other proteins containing the SAM domain, we generated a predicted model of the Ack1 SAM domain using AlphaFold [86,87]. The Ack1 SAM domain prediction adopts a typical SAM domain fold, which comprises four short α-helices (α1–α4) and a long C-terminal α-helix (α5) (Figure 6A). The predicted SAM domain is highly similar to that of EphA4 (Figure 6B), which is consistent with the sequence similarity between the SAM domains of EphA4 and Ack1 (Figure 6C). The consensus sequence for the two SAM domains contains two conserved hydrophobic buried residues (Trp and Leu) (Figure 6C, box 1), the Glu-Asp-Leu sequence of α3 (Figure 6C, box 4), followed by the Ile-Gly-X of the α3–α4 loop. These buried residues fall within the hydrophobic helix-helix interface. Furthermore, the boxed residues (Figure 6C, boxes 1–7) are evolutionarily conserved in homologs of Ack1 in other species, highlighting their structural importance.

**Figure 7:**
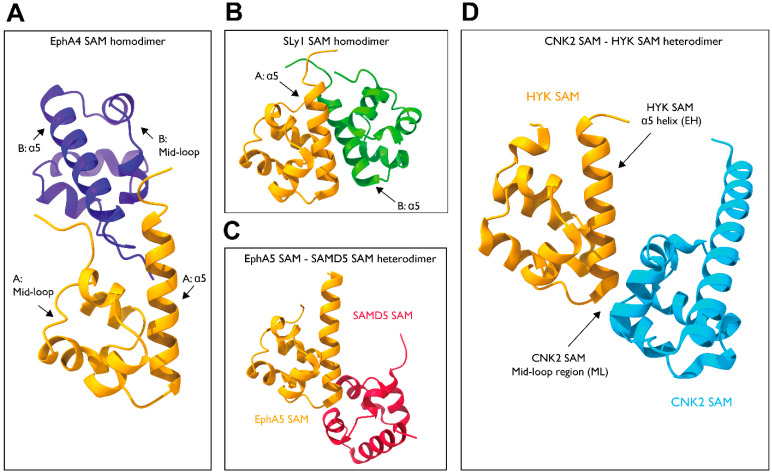
Structure of the SAM–SAM dimers. (**A**) EphA4-SAM homodimer (PDB: 1B0X). (**B**) SLy1-SAM homodimer (PDB: 6G8O). (**C**) EphA5-SAMD5 dimer (PDB: 5ZRZ). (**D**) CNK2-HYP dimer (PDB: 3BS5).

The EphA4 forms a homodimer in which the N-terminal coil region in one SAM domain clasps with a C-terminus of the α5-helix in the other SAM domain, forming the main dimer interface (PDB: 1B0X). In addition, the α1 and α3 side chains interact with each other, aiding dimerization (Figure 7A) [88]. The SH3 domain-containing protein expressed in lymphocytes 1 (Sly1) also exhibits dimerization mediated by its SAM domain (PDB: 6G8O). Its structure reveals a dimerization mechanism in which the C-terminus and α5-helices of the two SAM domains are in contact with each other, forming a symmetrical dimer (Figure 7B) [89].

In addition to their role in controlling self-assembly, the SAM domains also mediate several signaling cascades through hetero interactions with the SAM domains of other proteins. For example, EphA5 and SAMD5 interact with each other through their SAM domains (Figure 7C) [90]. The crystal structure shows a binding mechanism in which the N-terminus of the α5-helix of EphA5 interacts with the mid-loop region of SAMD5 (PDB: 5ZRZ). Similar interactions are observed for EphA2/SHIP2 and EphA6/Odin complexes. Other examples include CNK2 and HYP (PDB: 3BS5), which dimerize through their SAM domains to mediate the RAF signaling cascade (Figure 7D). The SAM domains of CNK2 and HYP adopt end helix (EH) and mid-loop (ML) binding modes [91], respectively, as observed in EphA5 and SAMD5. 

The SAM–SAM domain interactions do not follow a universal blueprint. Given that the SAM domain of Ack1 is required for full activity and that formation of homodimers activates Ack1, the SAM domain of Ack1 could likely adopt a dimerization mechanism similar to that of EphA4, as evidenced by their similarity in secondary structure and sequence.

#### 2.1.2. Other Protein Kinases with SAM Domains

All Ack family kinases possess a SAM domain. The sister kinase of Ack1 in humans, TNK1, also has a predicted SAM domain. The SAM domain of TNK1 is postulated to have a similar role to that of Ack1 as deletion of TNK1 SAM leads to a significant decrease in TNK1 autophosphorylation (Y277) in cells [92]. This observation is paralleled by a large reduction in in vitro activity of TNK1. The same study postulated that the SAM domain of TNK1 is important for self-association in vitro and showed that the reduction in self-association was correlated to diminished catalytic activity [92].

In addition to the Ack family, the two other protein kinases known to possess SAM domains are the Eph family RTKs and the sterile alpha motif and leucine zipper-containing kinase ZAK, a member of the STK/MAP3K family. The SAM domain of ZAK aids in the formation of homodimers or oligomers in mammalian cells [93]. Although little is known about the role of the SAM domain in the regulation of ZAK, functional analysis of a disease-associated mutation occurring in the SAM domain of ZAK reveals that the residues in the hydrophobic core of this domain are crucial for proper folding, as mutations in this region strongly destabilize the ZAK SAM domain [94]. Interestingly, the SAM domains of Ack1 and ZAK share sequence similarity in their buried hydrophobic residues, despite being unrelated (Figure 6C).

Analogously, the hydrophobic core of the Eph receptor SAM domains is similar in all members of the Eph family. Although the SAM domains of these receptors are similar in their secondary structures, each of them interacts with a different set of SAM domains from other proteins, owing to the diversity in their primary structure. This exemplifies the wide range of interactions SAM domains can provide even within the same TK family. 

It should be noted that the SAM domains of Eph RTKs diverge from that of Ack1 regarding their location. While the SAM domain of Ack1 falls at the N-terminus of the kinase, Eph receptors carry C-terminal SAM domains. These features add an extra level of complexity to putative SAM–SAM dynamics in Ack1 and underline additional mechanisms that may be present that surpass the limitations of models based on primary and secondary structures.

### 2.2. Kinase Domain

The kinase domain of Ack1 is sandwiched between the SAM domain at the N-terminus and the SH3 domain at the C-terminus. At the structural and sequence levels, the kinase domain has characteristics that are highly conserved among other NRTKs. 

**Figure 8:**
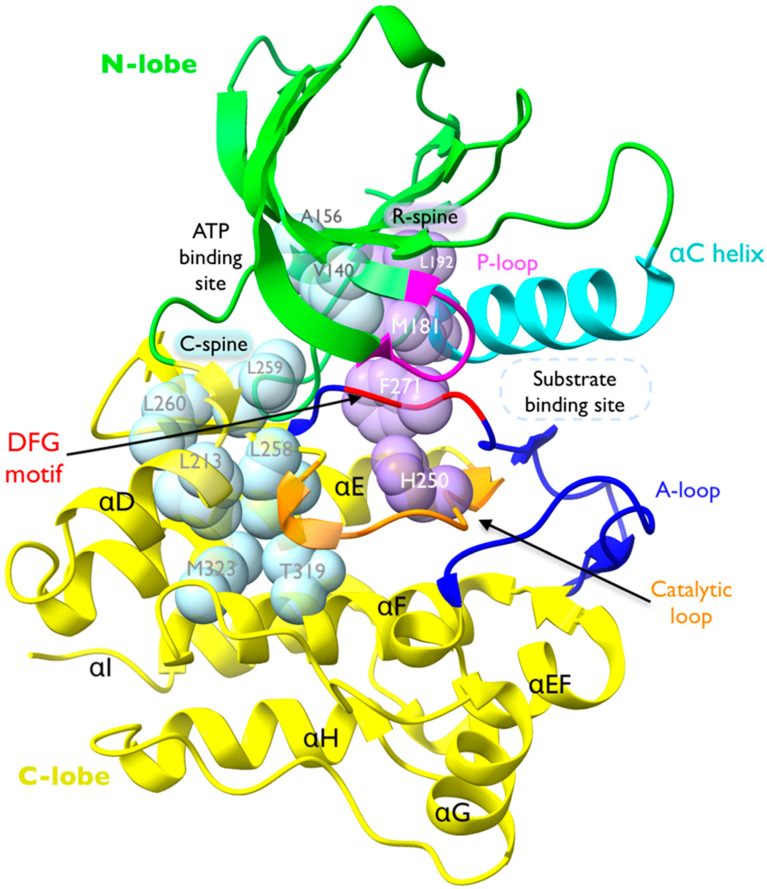
Structure of Ack1 KD. Crystal structure of active Ack1 (PDB: 4EWH). The bi-lobal fold of the kinase domain is highlighted with the N-lobe in green and the C-lobe in yellow. The universally conserved αC-helix is shown in cyan. The phosphate-binding loop (P-loop) is colored magenta. The activation loop (A-loop, blue) encompasses the DFG motif (red), which contributes to ATP binding. The catalytic loop is shown in orange. The residues comprising the catalytic spine (C-spine) and the regulatory spine (R-spine) are shown in light blue and light purple space-filling sphere models, respectively.

The Ack1 KD adopts a bi-lobed structure (Figure 8), which is typical of NRTKs and comprises an N-terminal lobe (green) and a larger C-lobe (yellow). The N-lobe contains an α-C helix (cyan) and β-sheet with a disordered nucleotide-binding loop that connects the C-term of β1 and the N-term of β2 (P-loop; magenta). The number and length of β-strands vary between different kinases, but the overall structure is conserved. A hinge connects the N- and C-lobes. The C-lobe comprises seven α-helices (αD to αl) and several β-strands with a catalytic loop (orange) that contains the HRD motif (also known as the catalytic triad), and an activation loop (A-loop; blue). While the catalytic loop connects αF to a β-strand, the A-loop connects a β-strand and α-EF [30]. The A-loop contains the DFG motif (highlighted in red), which points toward the ATP-binding site (Figure 8).

**Figure 9:**
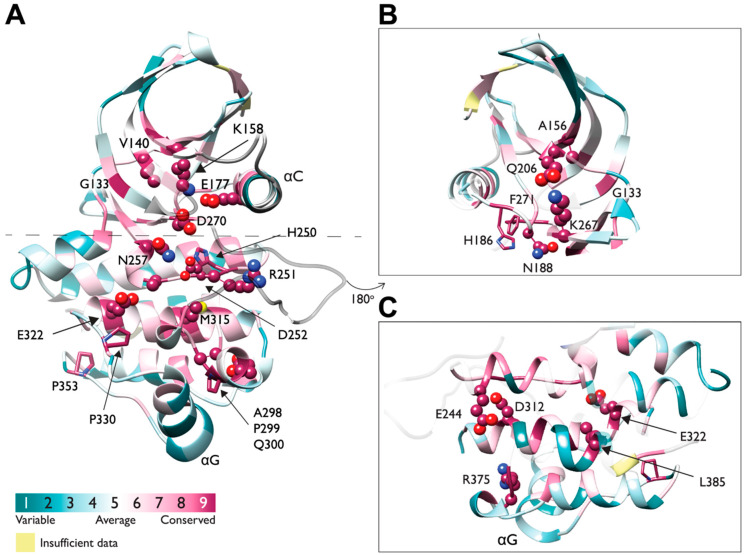
A 3D representation of evolutionarily conserved residues in Ack1 KD. (**A**) Sequence conservation of Ack1 KD presented as a cartoon diagram from two viewpoints (panel (**A**) rotated by 180° as panels (**B**,**C**)) and determined using the ConSurf server [96,97,98,99,100,101] using the crystal structure of active Ack1 (PDB: 4EWH) as the input. ConSurf assigns each residue with a normalized score of 1 to 9, where 1 indicates the least conserved (highly evolving) sites, and 9 corresponds to the most evolutionarily conserved positions. The conservation scores were mapped onto the Ack1 KD structure (PDB 4EWH) using ConSurf color code, with a cyan-to-maroon gradient corresponding to the variable (1) to conserved (9) positions. The panels depict conserved residues (with a score of 8 or 9) in the space-filling model. (**B**) The N-lobe rotated by 180° from panel (**A**). (**C**) The C-lobe rotated by 180° from panel (**A**).

The D270 of the DFG motif and the H250-R251-D252 comprising the catalytic triad are highly conserved (Figure 9A). Other conserved residues of the N-lobe include the K158 of β4 and E177 of αC-helix forming a salt bridge that is highly conserved throughout the entire protein kinase family, hallmarking the active state. The K158 belongs to the AxK motif, also conserved within Ack1 as A156-V157-K158 (Figure 9A,B), and interacts with the α and β phosphates of ATP [95]. Similarly, conserved residues within the C-lobe are mostly charged residues (Figure 9C).

The ATP-binding site is a highly conserved binding site for ATP and ATP-competitive inhibitors; a gatekeeper residue is often located within this pocket. Gatekeeper residues regulate the binding of nucleotides and influence the specificity of small-molecule inhibitors. This residue is often threonine, as evidenced in Abl and Ack1. Mutation of the gatekeeper residue is often linked to drug resistance, and T315I of Abl is a well-known example.

Another structural hallmark of a TK is presence of two hydrophobic spines called the catalytic spine (C-spine) and regulatory spine (R-spine). The R-spine is made up of two residues in the N-lobe and two in the C-lobe, which consist of the His of the HRD motif (H250), the Phe of the DFG motif (F271), M181 at the C-terminus of the αC-Helix, and L192 at the beginning of β4 (Figure 8 and Figure 9A,B). The assembly of the regulatory spine provides intramolecular interactions that define an active kinase and maintain kinase activity, while in the inactive structure, the R-spine is disassembled. Therefore, these residues are highly conserved.

The “catalytic spine” incorporates the adenine ring of ATP and establishes a connection between the N-lobe and the C-lobe upon nucleotide binding [102]. The residues in the catalytic spine are located in helix αF (M323 and T319) and helix αD (L213 and L258) in the C-lobe, and the N-lobe: L260 and L259 in the β7 strand, V140 in β2 strand, and A156 in β3 strand (Figure 8). 

In most protein kinases, the activation segment starts with the DFG motif (D270-F271-G273 in Ack1) and ends with an APE motif. The APE motif is present in human TNK1, mouse Ack1, and in the most distant (Figure 2) Ack1 relatives *C. elegans* Ark-1 and *D. melanogaster* Ack-like, but in human Ack1 this motif has been replaced with an APQ motif (A298-P299-Q300) (Figure 9A).

#### 2.2.1. Structure of the Active vs. Inactive Ack1 KD

Of the 12 structures available on the PDB, 11 capture Ack1 KD in its active form. Among these structures, 4EWH is the only structure with a resolved loop before the αC-helix [103], 4HZR has the best resolution [104], 4ID7 resembles the ATP-bound structure the most [80], 1U54 is the Y284-phosphorylated structure, and 1U46 is the unphosphorylated structure crystallized parallel to 1U54 [30]. 

Similar to other kinases, there are two hallmarks of Ack1 activation: a change in the (1) DFG motif conformation and (2) αC helix position. In the active state of Ack1, the conserved DFG motif is positioned such that D270 points toward the ATP-binding pocket, serving as a coordinator for divalent cations along with N257, with F271 facing toward the hydrophobic core. Meanwhile, the activation loop extends outward, creating a cleft for the incoming substrate. Upon substrate binding, N257 orients the catalytic residue D252, which serves as a nucleophile that targets the hydroxyl group of the substrate. The αC-helix exhibits an inward position, where a conserved E177 of the αC-helix forms a salt bridge with the activating lysine, K158, on the β-strand. The salt bridge orients K158, allowing it to form hydrogen bonds with the incoming ATP [30]. A conserved GxGxxG motif in the nucleotide-binding loop stabilizes the γ-phosphate of the ATP. Similar to other NRTKs, part of this motif (residue 135–138) and the loop connecting the N-terminal β-sheet and the αC-helix are disordered. 

Disruption of one of the two hallmarks deactivates Ack1. In the inactive crystal structure of Ack1 (PDB: 4HZS), the KD is autoinhibited by the SH3 domain, with four Ack1 KD molecules forming an asymmetric unit. Like other NRTKs, inactive Ack1 features the αC-helix moving outwards, breaking the salt bridge between K158 and E177. The E177 points away from the kinase core and forms a salt bridge with R275, stabilizing the outward conformation of the αC-helix. In addition, the β-sheet is rotated relative to the C-terminal lobe pivoted around the gatekeeper residue T205. The A-loop of inactive Ack1 flips, positioning the DFG motif to point away from the ATP-binding site. Repositioning of the αC helix with respect to the β-sheet can potentially affect Ack1 dimerization ability [30]. 

The two ligand-bound structures of Ack1 KD, one to debromohymenialdisine (PDB: 1U4D) [30] and another to 6-[4-[2-(dimethylamino)ethoxy]phenyl]-N-(1,3-dithiolan-2-ylmethyl)-5-phenyl-7H-pyrrolo [2,3-d]pyrimidin-4-amine (PDB: 4EWH) [103], exhibit similarities to those of CDK2 in its binding interactions. The CDK2 binds to the ATP competitive inhibitor hymenialdisine (PDB: 1DM2) through three hydrogen bonds between the inhibitor and residues E81 and L83, as well as several van der Waals interactions with the side chains of residues in the vicinity of the ATP-binding pocket [105]. The Ack1 forms hydrogen bonds with ATP-competitive inhibitors via A208 and D134. The hydrogen bond between D134 and the inhibitor stabilizes the nucleotide-binding loop, causing the loop to be well-ordered. 

#### 2.2.2. Ack1 KD in Comparison to Other TKs

Of all the TKs, the kinase domain of Ack1 is most closely related to EGFR KD. The two kinases share 39% sequence identity and have an RMSD of 1.1 Å with 237 of 252 possible Cα pairs superimposed [30]. Neither Ack1 nor EGFR requires phosphorylation for activation, which has little effect on kinase activity [106]. In both kinases, the A-loop is stabilized by hydrogen bonds between Arg, Asp, and Pro, allowing it to maintain a conformation resembling its phosphorylated active form in the absence of other co-factors and nucleotide-binding [102]. Similar to EGFR, activation of Ack1 requires dimerization independent of phosphorylation [84]. In addition, the region of the N-lobe involved in dimerization is nearly identical. However, instead of a symmetric dimer, EGFR forms an asymmetric homodimer where the N-terminus of one kinase is packed against the C-terminus of the other [107].

Similar to the structure of EGFR KD [108], the unphosphorylated and phosphorylated kinase domain structures of Ack1 KD show more of the characteristics of active kinases. However, there are some small differences inside chain conformations. In the phosphorylated state, phospho-Y284 is surrounded by one lysine and four arginines, three of which chelate the phosphate group of tyrosine and create an unusually electropositive environment around the tyrosine. Indeed, in most kinases, only one basic residue serves this function. The difference in electropositivity may be responsible for substrate binding and selectivity, while phosphorylation may be important for creating an effective electrostatic environment for catalysis [104]. A similar phenomenon is observed in another tyrosine kinase, v-Fps, where phosphorylation of the A-loop tyrosine (Y1073) enhances kinase activity only by 2-fold, and where the phosphotyrosine is chelated by two arginines rather than one [109]. 

### 2.3. SH3 Domain

The SH3 domain-containing proteins are found in multiple organisms, including eukaryotes and viruses. These include signal-transducing adaptor proteins and proline-rich tyrosine kinases. The SH3 domains interact with proline-rich regions and play a role in substrate recognition, autoinhibition, and cellular localization through protein–protein interactions. 

The NRTK families with SH3 domains include the Abl, Ack, Csk, Frk, Src, and Tec families [110]. The SH3 domain of these kinases is important for maintaining catalytic function. For example, the kinase activity of c-Abl and BCR-Abl is negatively regulated by the interaction between the SH3 domain and proline-rich regions [111,112]. The Csk family homodimerizes through interactions between the SH3 domains [113,114]. Inactive Src and Brk are stabilized by their SH3 domains [115], which also promotes substrate recognition [116].

Unlike typical NRTKs, the Ack family has a unique domain arrangement; instead of the typical N-terminal SH3 followed by the catalytic domain, the Ack family has a SH3 domain located at the C-terminus of the kinase domain. 

#### 2.3.1. Structural Features of the Ack1 SH3 Domain

**Figure 10:**
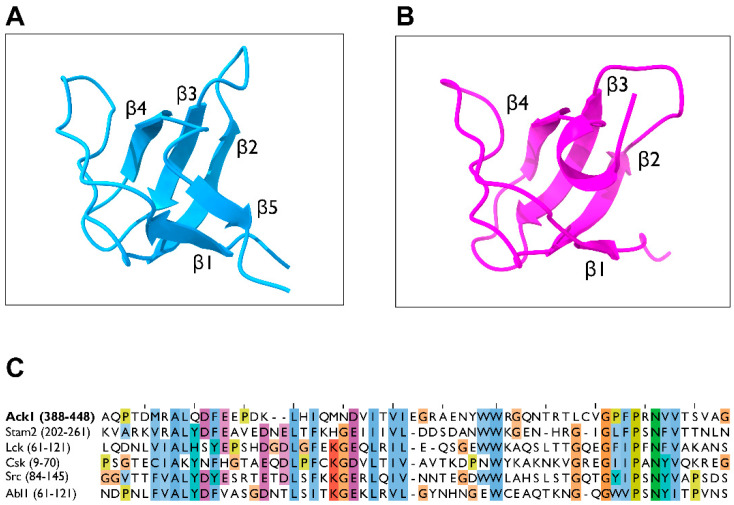
Structure of the SH3 domain. (**A**) Crystal structure of the Abl2 SH3 domain in the ribbon diagram (PDB: 5NP3, light blue). (**B**) Structure of the Ack1 SH3 domain (PDB: 4HZS, magenta). (**C**) Sequence alignment of the SH3 domains of human Ack1, Stam2, Lck, Csk, Src, and Abl. The numbers in parentheses denote the residues used as the inputs.

The SH3 domain of Ack1 adopts a structure similar to a typical SH3 domain of an NRTK, consisting of five-stranded β-barrel (β1–β5). Three β-strands (β2–β4) form an antiparallel β-sheet, orthogonally packed against the other antiparallel β-sheets (β1, β2, and β5), and separated by four loops (RT loop, n-Src loop, distal loop, and 3_10_ loop) [104]. The Ack1 SH3 domain shares this fold, except that it has a four-stranded β-barrel in which β-5 is replaced with an alpha-helix [104] (Figure 10). Structurally, the SH3 of Ack1 is most closely related to the SH3 of Lck (PDB: 2IIM) with an RMSD of 1.15 Å between 52 equivalent Cα atoms. 

The structure of the Ack1 kinase-SH3 domain construct (PDB: 4HZS), exhibits a dimer packed in a manner that allows each SH3 domain to be in contact. Based on this structure, the SH3 domain of Ack1 may interact with itself or with other SH3 domains. The interfacial residues of the SH3 domain that would allow for Ack1 SH3 homodimers are not conserved within the Ack family [104]. Therefore, it is unlikely that the Ack1 SH3 could form such homodimers as those observed in Csk where homodimerization is solely facilitated by the SH3 domain. 

#### 2.3.2. Potential Roles for the Ack1 SH3 Domain

The SH3 domains typically bind to the proline-rich sequence PxxP, where x denotes any amino acid, as evidenced by the Csk and Src family kinases [113,117,118]. Likewise, the SH3 domain of Ack1 binds to proline-rich regions of other proteins, such as the HSH2 adaptor protein in hematopoietic cells [119], and aids the phosphorylation of HSH2 by binding to its N-terminal proline-rich region. A similar phenomenon is observed in other kinases: Fyn kinase is activated by binding of Sam68 to its SH3 domain [120]. The SH3 domain of Lck binds to c-Cbl both in vivo [121] and in vitro, participating in downstream signaling events [121,122].

Notably, the SH3 domains can also bind to ligands without the PxxP sequence. For example, the SH3 domain of adaptor protein Grb2 serves as a competitive switch where the binding site of the non-PxxP motif domain overlaps the PxxP motif-binding site [123]. The ability of a SH3 domain to bind a non-PxxP motif can allow for special protein–protein assembly mechanisms in which a PxxP motif binding site and a non-PxxP motif binding site bind simultaneously. Such mechanisms are identified in *Saccharomyces cerevisiae* in the assembly of PEX13p–PEX14p–PEX5p docking proteins [124]. There are additional examples of SH3 domains showing a preference for different motifs. The SH3 of Stam2 favors interactions with RxxK and Px(V/I)(D/N)RxxKP sequence containing peptides [125,126]. Considering the high SH3 similarity between Ack1 and Stam2, the SH3 of Ack1 may also be involved in signaling cascades by interacting with both PxxP and non-PxxP peptides, underlining the potential of Ack1 to participate in the diverse binding behaviors.

Interestingly, Ack1 contains a proline-rich region in the C-terminal region within the MHR (Figure 4). The SH3 of Ack1 is proposed to interact with this region and facilitate autoinhibition [2] or regulate catalytic activity in a manner similar to that of Abl [111]. This phenomenon is exemplified by the mutations in the SH3 domain of Ack1 resulting in hyperactivation of its KD [51]. Other examples of SH3-mediated autoinhibition include the NRTK BCR-Abl [111], Src [127], serine/threonine kinase MLK3 [128], and NCF1 [129]—a subunit of neutrophil NADPH oxidase.

### 2.4. CRIB Domain

**Figure 11:**
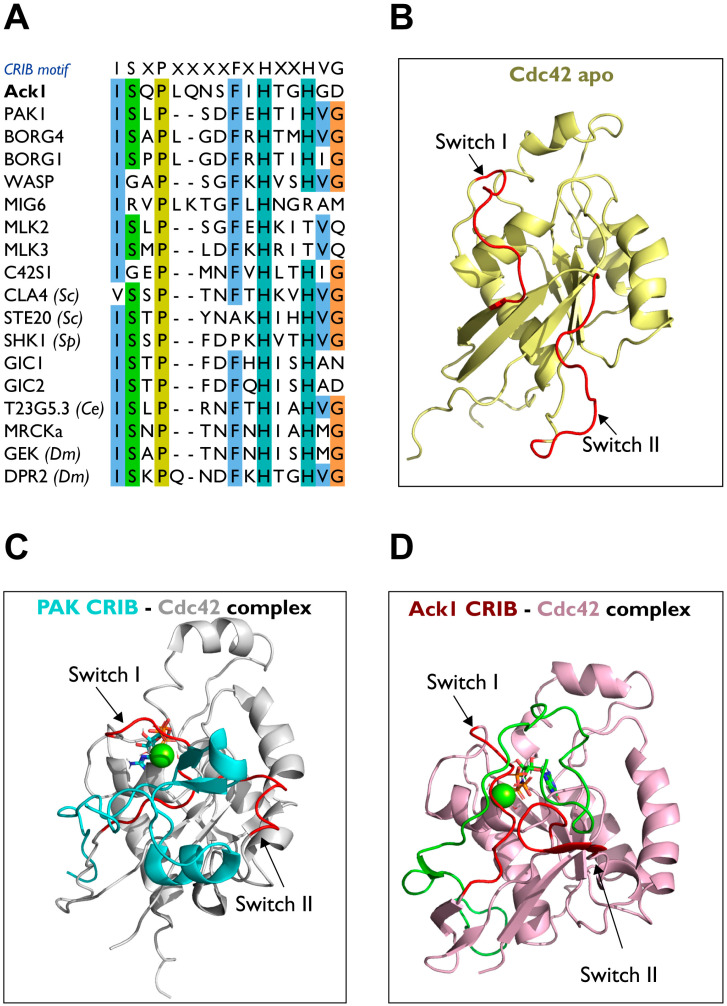
Structure of the Cdc42-CRIB complex. (**A**) Sequence alignment of the CRIB motifs of human Ack1 and other proteins. Sc, *Saccharomyces cerevisiae* (budding yeast); Sp, *S. pombe* (fission yeast); Dm, *D. melanogaster*; Ce, *C. elegans*. (**B**) Structure of apo-Cdc42 (PDB: 1AJE; pale yellow). (**C**) The PAK-Cdc42 complex (PDB: 1E0A, PAK CRIB, cyan; Cdc42, grey). (**D**) Ack1-Cdc42 complex (PDB: 1CF4; Ack1 CRIB, green; Cdc42, pink). Switches I and II are highlighted in red.

Effector proteins downstream of the small GTPases Cdc42 (or Rac) contain a GTPase-binding domain (GBD) that interacts with Cdc42 in its active GTP-bound form. The Cdc42/Rac interactive binding motif (CRIB) is the most conserved region within the GBD. CRIB motif comprises approximately 16 amino acids with the consensus sequence ISxPxxxxFxHxxHVG [56] (Figure 11A).

Although the CRIB motif is necessary for the binding to Cdc42 and Rac, it is not sufficient to give a high-affinity binding [130]. A less conserved inhibitory switch (IS) domain located at the C-terminus of the CRIB motif is responsible for maintaining proteins in a basal (autoinhibited) state [131,132,133].

Human Ack1 and its homologs are the only tyrosine kinases known to have a CRIB domain [134]. The activated mammalian serine/threonine kinase p21 kinases, PAK1–4 [135], and their homologs STE20 and CLA4 in yeast carry CRIB motifs [136]. Similar to Ack1, the PAK family kinases are downstream effector proteins of the Rho GTPase Cdc42. However, unlike Ack, PAKs additionally bind to the other Ras-related Rho GTPase, Rac. The MRCK and MLK family of kinases in *Drosophila* and humans also possess a CRIB domain. Other mammalian proteins such as the Wiskott–Aldrich syndrome proteins (WASP) involved in actin cytoskeletal rearrangements, contain CRIB domains, and are phosphorylated by Ack [5].

Strikingly, Mig6 is among the signaling proteins that possess a CRIB domain. Evolutionary analysis of the eukaryotic CRIB-containing proteins suggests that both Ack1 and Mig6 have CRIB domains most closely related to CEP4 [136]. The Ack1 and Mig6 also share sequence homology at the C-terminus of the Ack1 CRIB domain within the MHR.

#### Structural Comparison of CRIB of PAK vs. Ack1

As a Cdc42 effector, Ack1 mediates Cdc42-induced cell migration by modulating p130Cas signaling through Cdc42-CRIB domain interactions. The Cdc42 is comprised of nine β-strands and five α-helices. Furthermore, Cdc42 contains two switch motifs, one between α1 and β2 (switch I) and another between β3 and β4 (switch II) (Figure 11B). The binding behavior of the CRIB domain of Ack1 to Cdc42 is similar to that of PAK (PDB: 1E0A) (Figure 11C) [137]. The PAK binds between switches and helices α1, α5, and β2 strand of Cdc42 through the conserved phenylalanine and histidine residues of PAK-CRIB. The two switches change their positions to accommodate the binding of the CRIB domain (Figure 11D) [5].

The Ack1 forms an intermolecular β-sheet with Cdc42 by binding to the β2 strand of Cdc42 (PDB: 1CF4), while Y40 of Cdc42 interacts with F518/H520. In addition, S511 of Ack1 interacts with I46 and G47 of β2, and packs I173, and L177 in the αC helix (Figure 11D) [5].

In the PAK-Cdc42 complex, the intermolecular β-sheet between Y40-I46 of Cdc42 and I75-H83 of PAK contains Y40 of Ccdc42 interacting with F81/H83 of PAK. A few residues outside the CRIB domain also contribute to the binding; L543 and L541 of Ack1; L106 and L107 of PAK interact with L67 and L70 (switch II) of Cdc42 [130,132,137]. These leucines are crucial residues for the activation of PAK [138] and the activation of Ack1 [130,139] through the binding to GTP-bound Cdc42.

There are some minor differences in the binding behaviors of PAK-CRIB and Ack1-CRIB to Cdc42. Unlike PAK-CRIB, the α-helix of Ack1-CRIB forms a right angle to switch II upon binding, while in PAK-CRIB, the α-helix binds to residues 62–70 of Cdc42. In addition, Ack1-CRIB forms a more regular intermolecular β-sheet with the β2 strand of Cdc42. The buried surface of Ack1-CRIB is ~4200 Å^2^, compared to ~2500 Å^2^ in PAK-CRIB. PAK-CRIB forms a β-hairpin structure that binds to the top of switch II, while Ack1-CRIB binds to switch II through a loop. This difference in binding behavior may contribute to the difference in affinity between PAK-CRIB vs. Ack1-CRIB for Ras-related Rho GTPase Rac [5,135].

### 2.5. Clathrin-Binding Motif

Except for TNK1 and *Drosophila* DACK, Ack family kinases are clathrin-binding (CB) proteins. The Ack1 can directly associate with the heavy chain of clathrin and localizes to clathrin-containing vesicles [6,20]. Overexpression of Ack1 can disrupt clathrin distribution in an MHR-dependent manner.

The clathrin-binding motif LIDFG drives an interaction involved in receptor-mediated endocytosis. The Ack1 participates in receptor trafficking through its CB motif and downregulates EGFR [19,25,140]. Interestingly, an endocytic adaptor protein in yeast, Sla1p, binds to clathrin through a SAM domain and oligomerizes. Mutations in the CB motif and mutations that block oligomerization lead to endocytosis defects in Sla1p [83]. This raises the question of whether the SAM domain of Ack1 might have a potential role in clathrin-mediated endocytosis.

#### Ack1-Mediated Regulation of EGFR Trafficking and Degradation

Clathrin-mediated endocytosis is the major internalization pathway for many cell surface receptors, including EGFR. The Ack1 has significant regulatory roles in EGFR endocytosis, trafficking, and sorting for lysosomal breakdown [7,20,21]. The Ack1 binds to regulators of vesicle trafficking including AP2, clathrin, and sorting nexin 9. The Ack1 has been shown to regulate EGFR degradation, leading to down-regulation of EGF signaling. The Ack1 gene knockdown decreases ligand-induced EGFR endocytosis and degradation. Following EGF stimulation, Ack1 was found to colocalize with EGF on early endosomes. The Ack1 interacts with ubiquitinated EGFR to promote EGFR degradation via a process requiring phosphorylation of the Arp2/3 regulatory protein, cortactin. A somatic mutation in Ack1 that prevents ubiquitin binding can maintain the EGFR at the plasma membrane [141], prolonging mitogenic signaling following EGF activation and making some malignancies resistant to EGFR kinase inhibitors such as gefitinib. High levels of Ack1 expression limits EGFR internalization, which might be due to clathrin aggregation [19].

### 2.6. MHR

**Figure 12:**
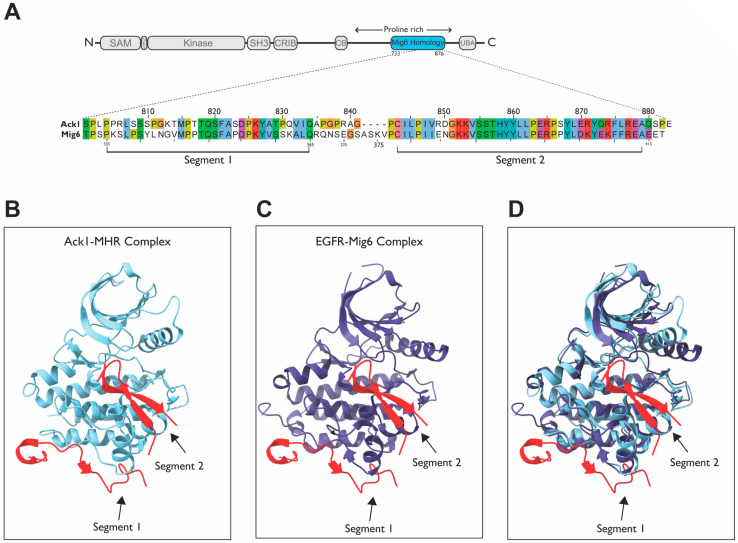
Sequence and structural comparison of Mig6 and MHR. (**A**) Sequence alignment between Ack1 and Mig6; (**B**) ClusPro predicted the Ack1-MHR complex [133,134] (PDB: 4EWH; Ack1, light blue; MHR, red); (**C**) EGFR-Mig6 complex (PDB: 4ZJV; EGFR, purple; Mig6, red). (**D**) Overlay of the Ack1-MHR and EGFR-Mig6 complexes from panels (**B**,**C**).

One of the unique features of Ack1 is its homology to the Mitogen-Inducible Gene 6 protein, Mig6, a negative feedback inhibitor of EGFR. The Mig6 protein inhibits EGFR activity by binding to its kinase domain and facilitating its endocytosis [142]. The residues through which Mig6 binds to EGFR are grouped into two segments (segments 1 and 2), corresponding to the two different EGFR interaction sites. Activated EGFR forms an asymmetric dimer in which one kinase acts as an activator of the other [107]. Segment 1 of Mig6 inhibits EGFR by blocking the formation of this activating dimer and binds to the dimer interface whereas segment 2 occupies the substrate-binding site. These segments are conserved within the MHR of Ack1 (Figure 12A).

Other than Ack1, no other protein has any homology to Mig6, suggesting that the high conservation of the residues might have functional significance. Strikingly, Ack1 and Mig6 are 80.6% homologous and 53.7% identical within the MHR (Figure 12A). Segment 2 of Mig6 is highly conserved, with nearly 75% sequence identity. This region binds to the substrate-binding site of EGFR; therefore, segment 2 of MHR might block the active site of Ack1 in the basal state (Figure 12B vs. Figure 12C).

**Figure 13:**
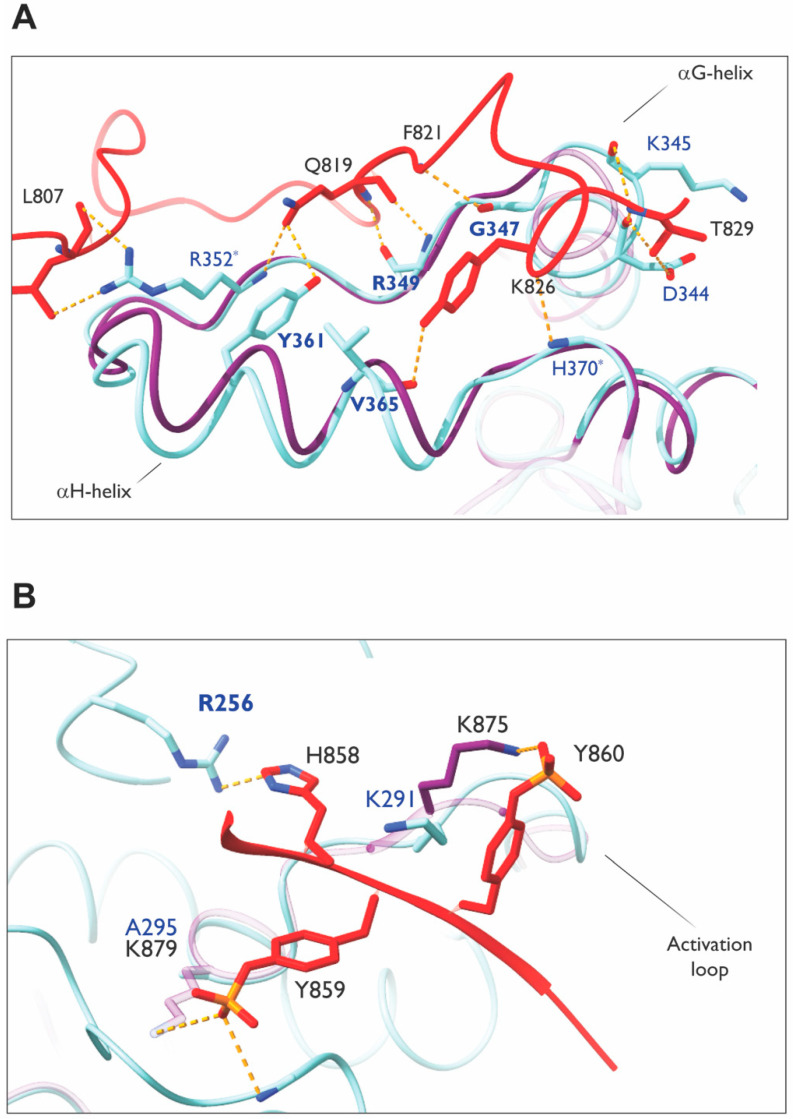
Homology modeling of MHR–Ack1 KD. (**A**) Model showing MHR segment 1 interaction sites with the C-lobe of Ack1 KD. The αH-helix and αG-helix are marked as reference points. Segment 1 of MHR (red) is putatively superimposed on Ack1 KD (light blue) using the crystal structure of EGFR KD (purple) bound to Mig6 (PDB: 4ZJV). The MHR residues are labeled in black; Ack1 KD residues are in navy. The conserved residues between EGFR KD and Ack1 KD are marked in bold. Non-conserved novel interacting partners are marked with an asterisk. (**B**) Segment 2 interaction sites at the substrate binding site. The activation loop is labeled as a reference point. Segment 2 of MHR (red) is superimposed on Ack1 KD (light blue) using the crystal structure of EGFR KD (purple) bound to Mig6 (PDB: 4ZJV). The MHR residues are labeled in black; Ack1 KD residues are in navy. Hydrogen bonds are depicted in yellow dashed lines. Note that a key lysine in EGFR KD that interacts with Mig6 is not conserved in the Ack1 KD.

The MHR of Ack1 has been proposed to have an auto-inhibitory role; a phenylalanine residue (F352) in segment 1, which plays a key role in the Mig6-EGFR interaction [143] (Figure 13A), is conserved in Ack1. Alanine mutation of this residue (F821A) was found to activate Ack1 by ~80 fold, rendering MHR unable to bind to the kinase domain [51]. The Ack1 also possesses residues analogous to those involved in Mig6 (segment 1)–EGFR interactions or a “segment-1 binding surface”, which could participate in autoinhibition. These findings are supported by other studies, suggesting that Ack1 is autoinhibited by intramolecular interactions through the MHR domain in the basal state [28,104].

As a region rich in prolines, MHR serves as a site for protein–protein interactions, especially for those with SH3 domains. These include interactions between EGFR and Ack1, which are mediated indirectly in a Grb2-dependent (through its SH3) manner or directly in an MHR-dependent manner. Other binding partners for proline-rich sequences within Ack1 include the NRTK Hck [106], the Heat shock protein HSP 90 kDa alpha proteins, HSP90AA1, and HSP90AB1 which interact with Ack1 via their SH3 domains. This interaction regulates the Ack1-dependent phosphorylation of STAT1 and STAT3.

#### Modeling of Interactions between the Segment 1–2 of MHR and Ack1 KD

Based on the crystal structure of the EGFR kinase domain and phosphorylated Mig6 encompassing both segments 1 and 2 (PDB: 4ZJV), Mig6 segment 1 binds to the bottom of the KD C-lobe independently of segment 2 (Figure 13A). Segment 2 occupies the substrate-binding site (Figure 12B–D). The two phosphotyrosines (pY394 and pY395) in segment 2 make favorable electrostatic interactions; the side chain of pY395 interacts with the K875 side chain, while the pY394 side chain interacts with the backbone atoms on the N-terminal residues of the αG helix and K879 side chain (Figure 13B).

A model superimposing segment 2 of Mig6 onto the KD (mimicking segment 2 of MHR) has a binding behavior similar to that of Mig6 towards EGFR (Figure 12B–D). However, Mig6 is a cytosolic protein that cycles between association and dissociation with EGFR (and other receptors), while MHR is a domain within Ack1, subject to intramolecular forces. Hence, the inhibitory interactions between Ack1-MHR may not necessarily be regulated by relatively strong interaction forces, such as those seen in EGFR.

The phosphorylation of Y394 and Y395 is critical for the successful inhibition of EGFR [144], as unphosphorylated segment 2 was found to have a very low affinity towards the KD. The interacting partners of Mig6 Y394-Y395 in EGFR KD are also conserved in Ack1 KD, with one exception. The lysine that coordinates pY395 of Mig6, K879, is replaced with alanine in Ack1 (A295). Tyrosines 394–395 of Mig6 correspond to Y859-Y860 of MHR in Ack1, which are suggested to be phosphorylated similarly [145].

The K875 of EGFR plays a crucial role in acting as a hinge in orienting phosho-Y395 of Mig6, which is catalyzed by Src, priming Y394 for phosphorylation by EGFR. Phosphorylated Y394 then can be readily coordinated by K879. Therefore, the presence of two lysines is a key element in orienting the two tandem tyrosines in EGFR KD–Mig6 interactions. Though these tyrosines are conserved as Y859 and Y860 in Ack1, only one lysine is present in the Ack1 kinase domain. Interestingly, K875–K879 of EGFR are also conserved in Abl (K419–K423), a tyrosine kinase that binds the two tandem tyrosines of Mig6. In contrast to EGFR, however, Y394-Y395-phosphorylated Mig6 activates Abl kinase activity by relieving inhibitory intermolecular interactions.

### 2.7. UBA

Polyubiquitylation and degradation are regulated by the interaction of the UBA domain with mono- or polyubiquitin chains [146]. Generally, an UBA domain facilitates three types of functions: (1) to act as a target site for ubiquitination such as in p62, where K420 of the UBA domain is ubiquitinated by Keap1/Cullin3 [147]; (2) to act as the binding site of ubiquitin chains, as in adaptor protein RAD23 [148]; and (3) to aid in protein–protein interaction through UBA domain homo- and hetero-dimerization, as observed in the Rad23 homodimers and Rad23/Ddi1 heterodimers [149].

In contrast to that of Ack1, the UBA domain of the other human Ack family kinase, TNK1, serves a positive regulatory role. The TNK1 is phosphorylated by microtubule affinity-regulating kinases (MARKs), resulting in the abolishment of its activity through the binding of 14-3-3 [12,150]. In the absence of MARK-mediated phosphorylation, ubiquitin binding to TNK1 activates TNK1 and promotes its movement to cytosolic ubiquitin clusters [12].

#### 2.7.1. Role of Ack1-UBA in Protein Stability

There are two pathways controlling the stability of Ack1: (i) through lysosomes [85] and (ii) through proteasomes [151]. The HECT E3 ubiquitin ligase Nedd4-1 has been found to drive EGFR-dependent Ack1 degradation via the lysosomal route and the SIAH binding motif at the C-terminus of Ack1 controls its SIAH-dependent turnover via the proteasomes. The Nedd4-2 requires Ack1 kinase activity to downregulate Ack1, whereas the SIAH proteins interact with and degrade ACK1 independent of kinase activity. The Ack1 interacts withs the ubiquitin ligase Nedd4-2 through a conserved PPXY motif, which is crucial for their colocalization in clathrin-rich vesicles. This motif is located near the N terminus of the UBA domain.

The Ack1 is involved in the transport of EGFR into a non-canonical degradative pathway [19]. The Ack1 localizes inside sequestosome-1 (p62/SQSTM1) and ubiquitin-rich compartments in unstimulated cells, and upon EGF stimulation, it localizes away from p62/SQSTM1 to early endosomes to promote non-canonical EGFR trafficking [140]. When stimulated by EGF, Ack1 is thought to localize to both early endosomes and autophagosomes. The UBA domain of Ack1 is required for association with p62/SQSTM. The deletion of the UBA domain significantly reduces this interaction and desensitizes it to EGF treatment.

Knockdown of Ack1 enhances EGFR trafficking to lysosomal compartments; the presence of Ack1 inhibits EGFR from rapidly translocating to lysosomes after EGF stimulation. It is important to note that deletion of the UBA domain of Ack1 has no effect on its association with EGFR, implying that ubiquitylation of EGFR is unnecessary for this association [25].

The interaction of EGFR with the clathrin-binding domains of Ack1, as well as Ack1 association with ubiquitin and p62/SQSTM, are thought to be the fundamental mechanisms for EGFR non-canonical trafficking. The Ack1 acts as a shuttle between the p62/SQSTM1 compartments and the canonical endocytic pathway, preventing EGFR trafficking via the classical lysosomal pathway.

#### 2.7.2. AlphaFold Modeling of the Ack1 UBA Domain

**Figure 14:**
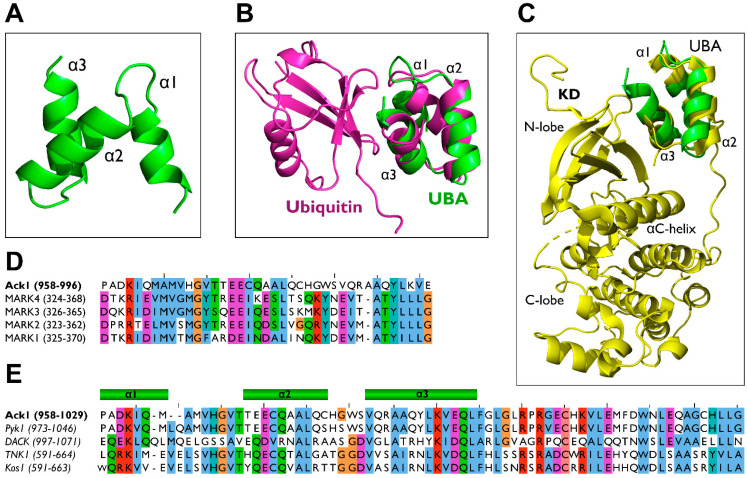
Ack1 UBA domain homology modeling. (**A**) Ack1 UBA domain structure predicted by AlphaFold [86,87] represented in a ribbon diagram (**B**) Structural alignment of predicted Ack1 UBA domain (green) and the yeast Ede1 UBA-ubiquitin complex (PDB: 2G3Q, magenta) (**C**) Structural alignment of predicted Ack1 UBA (green) and MARK3 UBA-KD complex (PDB: 2QNJ, yellow). (**D**) Multiple sequence alignment of the UBA domains of the human Ack1 UBA and MARK families. The numbers in parentheses denote the number of residues in each protein. (**E**) Multiple sequence alignments of the UBA domains of the Ack1 family: human Ack1, mouse Ack1/Pyk1, fly DACK, human TNK1, and mouse Kos1. The numbers in parentheses denote the residues used as inputs for the alignment.

The AlphaFold model for the UBA domain of Ack1 comprises three α-helices (Figure 14A). This generic secondary structure is common among the UBA domains of other proteins, such as p62 [152], PLIC1 [153], Mud1 [149], and AMPK family kinases [154,155]. Although the sequence among different UBA domains is not highly conserved, it contains a conserved tyrosine (Y992 of Ack1) on the third helix (α3), which forms π–π interactions with the first helix (α1) and stabilizes the UBA domain to maintain the conventional triple-helix fold [126].

Because the UBA domain of Ack1 is a target for mono-and polyubiquitin chains [113], it is plausible that the UBA domain of Ack1 adopts a ubiquitin-binding mechanism similar to that of other UBA domain-containing proteins (Figure 14B), in which α1 and α3 of the UBA domain interact with the hydrophobic I44-containing surface of ubiquitin [146,152,153,156].

During ubiquitin binding, the surface of discontinuous helices forms a contact interface with the β-sheet of ubiquitin [123]. Most UBA domains contain an MGF/Y motif at the end of α1 and a leucine residue at the end of α3 [124,126]. These conserved residues in the UBA domain are crucial for ubiquitin interactions. The Ack1 also contains Leu at the C-terminus of α3. However, instead of the MGF motif, vertebrate Acks have an HGV sequence (Figure 14D,E). This difference underlies the possibility of Ack1’s unique ubiquitin-binding mechanism.

#### 2.7.3. UBA in AMPK-Related Kinases: Functional Comparison

Besides Ack1, AMPK-related serine/threonine kinases (MARKs) have UBA-like domains that play a key role in the autoinhibition and activation of the kinase by binding to the kinase domain [154,157]. Unlike a typical UBA domain, the UBA domains of AMPK-related kinases have little or no interaction with ubiquitin and its analogs [157].

The UBA domain of MARK adopts the traditional fold of an UBA domain with a conserved MGY motif (Figure 14D) in the interhelical loop of the parallel helix. It similarly binds to ubiquitin as other UBA domains do, however, NMR studies suggest that binding of the MARK UBA domain to ubiquitin might involve other regions of the UBA domain [154].

The kinase domains of Ack1 and MARK3 exhibit increased kinase activity upon binding to their UBA domains. While there are minimal structural data regarding the Ack1 KD-UBA interaction, the crystal structure of MARK3 reveals a possible kinase activation mechanism mediated by the UBA domain (Figure 14C), where the UBA domain of MARK3 binds to the β-sheet region of the KD N-lobe and stabilizes the active conformation of the kinase [154].

Unlike Ack1, the UBA domain of MARK binds ubiquitin with minimal affinity [126]. This could be caused by the lack of conformational stability of the MARK3 UBA domain, in which the α3-helix of the UBA domain loses its helical feature in its free unbound state, which may be associated with low ubiquitin affinity.

## 3. Discussion

### 3.1. Unique Protein–Protein and Domain–Domain Interactions Governing Ack1

**Figure 15:**
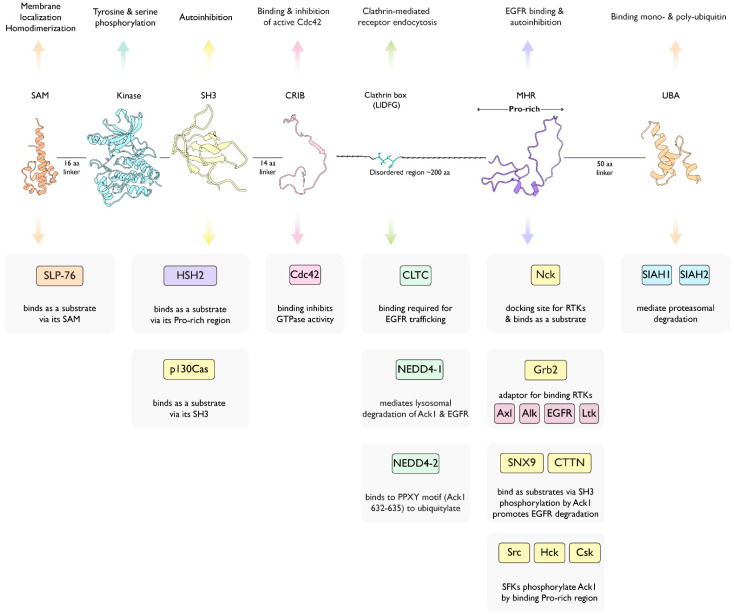
Graphical summary of known Ack1-protein interactions mapped onto a linear diagram of Ack1 domain structure. SAM, sterile alpha motif; SH3, Src homology 3; CRIB, Cdc42, and Rac-interactive binding; MHR, Mig6 homology region; UBA, ubiquitin-associated domain. Linker regions are designated disordered according to UniProtKB (Q07912) and AlphaFold prediction. Interacting proteins are color-coded according to the nature of the interaction such that the SH3-mediated are in yellow, the Pro-rich is purple, and the SAM-mediated is orange. Details of these interactions are as follows: SLP-76 [40], HSH2 [119], p130Cas [159], Cdc42 [134], Clathrin heavy chain (CLTC) [6], NEDD4-1 [85], NEDD4-2 [160], Nck [6], Grb2 [161], SNX9 [162], Cortactin (CTTN) [163], Src and Hck [106], Csk [164], SIAH1 [151], and SIAH2 [165].

Using the structures and AlphaFold-predicted models used in this review, we summarized the functions of domains in Ack1 and the protein–protein interactions they facilitate, indicating the partner domains responsible where possible (Figure 15).

The Ack1 is regulated by intermolecular and intramolecular mechanisms. The Ack1 forms dimers (or multimers) in cells. The SAM domain-mediated dimerization of Ack1 is necessary, but not sufficient for activation [84]. The MHR has been suggested to have an autoinhibitory role; it can also bind to the EGFR kinase domain [25]. This Ack1-EGFR interaction may play a role in regulating Ack1 activity. Phosphorylation of the C-terminal MHR disrupts autoinhibitory interactions [51,145] and could lead to a conformational change that allows membrane recruitment and dimerization by the SAM domain [84]. The interplay between SH3, MHR, and Pro-rich regions is also not completely understood; it has been suggested that the interaction between the SH3 and the Pro-rich region could position the MHR for inhibiting the kinase domain [2,158]. Of note, according to the UniProtKB and AlphaFold, the region connecting the CRIB domain to the C-terminus (MHR and UBA), of ~200 residues in length, is predicted to be disordered.

The Ack1 is the only protein known in the literature to carry a region highly homologous to Mig6, a negative feedback inhibitor of EGFR. The EGFR-Mig6 and Ack1-MHR interactions differ fundamentally in that Mig6 is a separate protein that regulates EGFR via association and dissociation, whereas MHR is a domain on Ack1 that presumably binds via intramolecular interactions. The entropic effect of being present on the same polypeptide would increase the affinity of the MHR for the kinase domain in full-length Ack1. As a result, the affinity of the Ack1 KD for the isolated MHR domain (or of peptides derived from the MHR) would be expected to be much lower than those of Mig6 for EGFR KD, which presents a challenge for experimentally addressing the Ack1 KD-MHR interactions.

The only other human tyrosine kinase known to carry an UBA domain in addition to Ack1 is its sister kinase TNK1. The UBA of TNK1 has been proposed to facilitate its oligomerization through interaction with other ubiquitylated proteins and locate TNK1 to ubiquitin condensates [12]. Andersen and colleagues also proposed that the UBA of TNK1 anchors it to substrates and might aid in the activation of the kinase. However, the UBA domain is dispensable for oligomerization such that TNK1 lacking the UBA retains autophosphorylation. Other domains on TNK1, such as the SAM and SH3 domains, may help the kinase self-associate [92]. The precise mechanism of TNK1 oligomerization remains to be elucidated. Future research questions that could be asked include whether or not TNK1 and Ack1 form hetero-oligomers.

### 3.2. Outstanding Research Questions for Understanding Basic Biology of Ack1

Out of all the existing Ack1 structures, only one autoinhibited Ack1 KD-SH3 structure has been reported. In addition to the SH3 domain, multiple studies have suggested a possible autoinhibition mechanism involving the MHR [51,104]. Hence, understanding the autoinhibited structure could shed light on the pathophysiology of prostate cancer, a malignancy for which significant implications of Ack1 have been heavily characterized.

The association of Ack1 with the wide range of protein–protein interactions and signaling cascades discussed above underlines the value of Ack1 as a potential therapeutic target. Further investigation of the structural characteristics and non-catalytic domains of Ack1 could provide insights into the development of promising selective drugs.

### 3.3. Emerging Research Questions of Physiological Relevance

Given the correlation between Ack1 dysregulation and tumorigenesis, the mechanistic details underlying the cross-talk of EGFR and Ack1 remains as an outstanding research question. The Ack1 regulates EGFR degradation and shares sequence homology with an EGFR feedback inhibitor while the kinase domains of EGFR and Ack1 share ~ 40% sequence homology and have closely related structures. Because their interaction facilitates internalization and degradation of EGFR, the status of Ack1-mediated signaling could have implications for EGFR-driven cancers such as glioblastoma, nonsmall-cell lung cancer, and colorectal cancer.

The Ack1 is involved in many biological functions including cell proliferation, migration, and apoptosis. Dysregulation of Ack1 is associated with multiple diseases such as cancer, neural disorders, and autoimmune diseases. However, more research is needed to elucidate the cellular functions of Ack1 distinct from those pertaining to cancer progression. For example, Ack1 was recently found to activate TLR signaling and promote the production of proinflammatory factors [40]. Future research should examine the involvement of Ack1 in immune cell signaling to pinpoint the molecular mechanisms underlying the observed phenotypes in autoimmune diseases.

A variant of Ack1 has been identified in three siblings with autosomal recessive infantile-onset epilepsy and intellectual disability [48], revealing a link between Ack1 mutations and neural disorders. The Ack1 has also been implicated in synapse formation, but the detailed mechanism remains unclear [17]. Molecular mechanisms linking Ack1 dysfunction to the phenotype in neural diseases require further investigation.

## Data Availability

Data are available upon request from the corresponding author.
